# Is human immunodeficiency virus (HIV) stage an independent risk factor for altering the periodontal status of HIV-positive patients? A South African study

**DOI:** 10.1186/1472-6831-13-69

**Published:** 2013-12-03

**Authors:** Cathy Nisha John, Lawrence Xavier Stephen, Charlene Wilma Joyce Africa

**Affiliations:** 1 Department of Periodontics and Oral Medicine, University of the Western Cape, Western Cape, South Africa; 2 Anaerobe group, Department of Medical Biosciences, University of the Western Cape, Western Cape, South Africa

**Keywords:** Periodontal disease, CD4 + T cell counts, Immunosuppression, HIV-positive patients, Age, Oral hygiene, Brushing, Interdental aids, HIV stage, ART, Smoking

## Abstract

**Background:**

The immunosuppresion in HIV patients makes them highly susceptible to microbial infections. The aim of the study was to establish whether HIV stage (as depicted by CD4+ T lymphocyte counts) could independently be associated with periodontal status (as revealed by the measurement of clinical indices).

**Methods:**

One hundred and twenty HIV-infected patients attending an infectious diseases clinic in the Western Cape, South Africa were included in the study. The periodontal clinical indices such as plaque index, gingival index, pocket probing depth and clinical attachment levels were measured on the mesial aspect of the six Ramfjord teeth. The CD4 + T cell counts were taken from the patients’ medical records and patients’ HIV stage determined and grouped according to their CD4+ T cell counts into A (<200 cells /mm^3^), B (200–500 cells /mm^3^) and C (>500 cells /mm^3^).

**Results:**

The mean age of 120 HIV-positive patients was 33.25 years and the mean CD4 + T cell count was 293.43 cells/mm^3^. The probing depth and clinical attachment loss were found to be significantly associated with the total CD4 + T cell counts but not with HIV stage. Significant correlations were found between age and all clinical indices except for clinical attachment loss. No correlation was found between age and HIV stage of the patients. The use of antiretroviral therapy was significantly associated with probing depth and clinical attachment loss, but not with plaque nor gingival index. Significant associations were observed between smoking and all of the clinical indices except for the gingival index. A significant association was observed between the use of interdental aids and all the clinical indices except for probing depth, while brushing was significantly associated with plaque index only. CD4 + T cell counts were significantly associated with brushing frequency (p = 0.0190) and the use of interdental aids (p = 0.0170).

**Conclusion:**

The findings of this study conclude that HIV stage, ART and age are not independent risk factors for changes in the periodontal status of HIV-positive subjects but rather that smoking and oral hygiene habits determine their susceptibility to disease.

## Background

Among the infectious diseases, human immunodeficiency virus (HIV) infection, which eventually results in acquired immune deficiency syndrome (AIDS), remains a global health problem. According to the United Nations AIDS global report [1] there are 34 million people living with HIV globally, with 5.6 million living in South Africa. The pathogenesis of HIV infection is considered to be related to the depletion or reduction of CD4 + T helper cells, causing profound immunosuppression which may predispose the individual to aggressive gingivitis or periodontitis unresponsive to conventional therapy [2]. Considered as one of the earliest clinical features of HIV infection [3], many studies have related the degree of immunosuppression and HIV viral load with the progression and exacerbation of chronic inflammatory periodontal disease (CIPD) [4,5], with prevalence and severity ranging from 1-66% for gingivitis and 0-91% for periodontitis [6,7].

Periodontal disease is an inflammatory disease with a multifactorial aetiology including immunological reactions to the dental plaque or oral biofilm. With the periodontium serving as a reservoir for microorganisms, other factors such as inadequate oral hygiene, smoking, age, and HIV stage (immunodeficiency) are recognized as risk factors which predispose an individual to periodontal disease [8-13]. Recently, the age concept has been challenged and it is considered that with proper oral hygiene maintenance, periodontal disease can be prevented [14].

The literature demonstrates varying degrees of susceptibility to periodontal diseases (75-80%) with 10% of the population appearing to be completely resistant, despite the presence of plaque [9]. It has been speculated that lack of oral hygiene and CD4 + T cell counts <400 cells/mm^3^ may reduce the ability of the host to control infection by periodontopathogens, resulting in CIPD [15-17].

The aim of the present study was to establish whether an independent relationship exists between HIV stage and periodontal clinical indices or whether other factors such as age, ART, smoking and/or oral hygiene habits may govern the prevalence and severity of periodontal disease in a cohort of South African HIV + patients.

## Methods

### Study population

A cohort of 120 randomly selected male and female HIV-positive patients attending the infectious diseases clinic at Tygerberg Medical Hospital, South Africa participated in the study. The study group included HIV-positive patients aged between 17–55 years of age, regardless of their CD4 + T cell counts or antiretroviral therapy status. Exclusion criteria focused on pregnancy, tuberculosis, diabetes, cardiovascular disease, autoimmune diseases as well as patients who had received antibiotic treatment or had undergone dental treatment 3 months prior to the study. The study was approved by the Research Ethical Committee from the University of the Western Cape. All patients were informed of the purpose of the investigation and written consent was obtained for participation in the study. Patients were assured of confidentiality and informed of their right to refuse to participate or withdraw from the study. Limited information regarding demographic features, general health, HIV infection history, CD4 + T cell counts, smoking and other predisposing factors to periodontal disease were obtained from questionnaires and patients’ medical records.

### Measurement of periodontal indices

Periodontal clinical measurements included plaque index (PI), gingival index (GI), probing depth (PD) and clinical attachment loss (CAL) as previously described [18]. These measurements were performed and recorded by a single calibrated examiner (kappa index and intra-class coefficient correlation of agreement for PD measurements = 0.765-0.985). We elected to assess the Ramfjord teeth which have been reported to provide a suitable alternative for full mouth examination [19] instead of the Community Periodontal Index of Treatment Needs (CPITN) recommended by the World Health Organisation (WHO) for epidemiological studies [20]. The reasons for this are further defined in the discussion.

### Statistical analysis

Data was analyzed using the SAS statistical programme (SAS Institute Inc., Cary, NC, USA). Besides frequency distribution, data analysis included Spearmans rank correlation and Wilcoxon tests. A significance level of <0.01 indicated a highly significant result, while results with a p-value between 0.01 and <0.05 were referred to as significant.

## Results

Table 1 shows the descriptive statistics of age, CD4 + T cell counts and periodontal indices of the 120 HIV-positive patients included in the study. The mean age was 33.25 years with a median age of 32 years (range: 20–55). The mean values for plaque index, gingival index, probing depth and clinical attachment loss were 2.55, 2.75, 4.77 mm and 5.29 mm respectively.

**Table 1:** **Mean** (**SD**) **and median of age**, **periodontal indices and CD4** + **T cell counts**

**Variables**	**n**	**Mean (SD)**	**Median**	**Minimum-Maximum**
Age (years)	120	33.25 (7.42)	32	20-55
Plaque index	120	2.55 (0.54)	2.8	0.8-3
Gingival index	120	2.75 (0.45)	3	0.5-3
Probing depth	120	4.77 (1.04)	4.9	2.9-6.8
Clinical attachment loss	120	5.29 (1.1)	5.35	3-7.3
Total CD4 + Tcell counts (A + B + C)	120	293.43 (151.06)	294.5	36-859
Grouped CD4 + T cells				
A (<200 cells/mm^3^)	36 (30%)	135.67 (37.44)	136	36-190
B (200–500 cells/mm^3^)	71 (59.17%)	321.27 (87.9)	312	200-500
C (>500 cells/mm^3^)	13 (10.83%)	578.24 (100.03)	534	510-859

**Figure 1:**
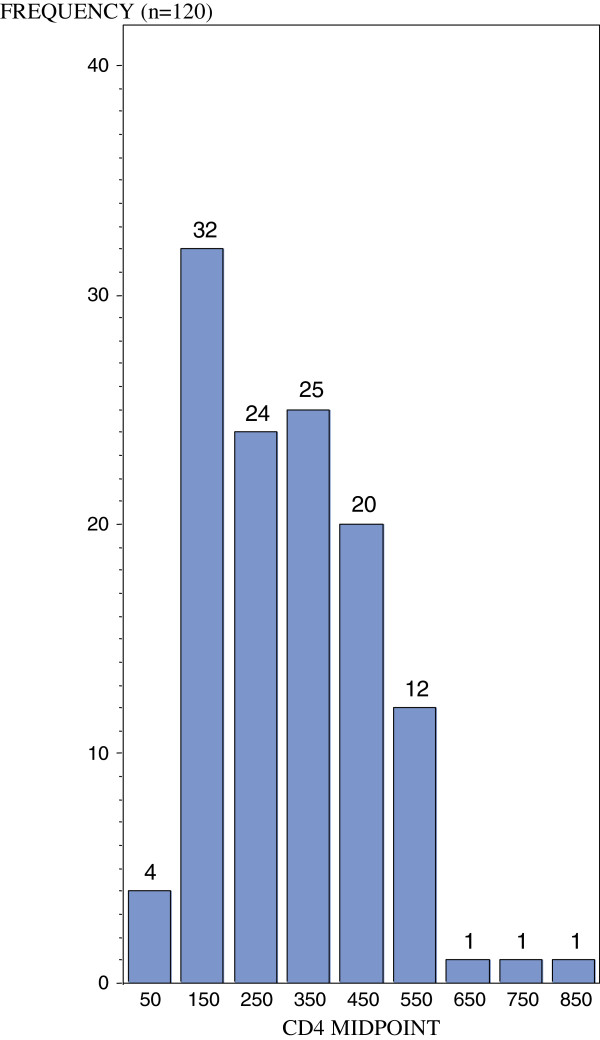
**Frequency distribution of CD4 + T cell counts grouped into intervals of 100 and plotted against the midpoint.**

The mean CD4 + T cell count was 293.43 cells/mm^3^ (Table 1). When grouped according to their CD4 + T cell counts into groups with <200 cells/mm^3^ (Group A), 200–500 cells/mm^3^ (Group B) and >500 cells/mm^3^ (Group C), 30% of the patients were assigned to Group A, indicating severe immunosuppression (Table 1), 59.17% to Group B, a moderate degree of immunosuppression, with Group C (11%) indicating a wide range of immune competence (510–859 cells/ mm^3^, Table 1). Figure 1 shows the breakdown of CD4 + T cell counts into intervals of 100, plotted against the CD4+ midpoint.

**Figure 2:**
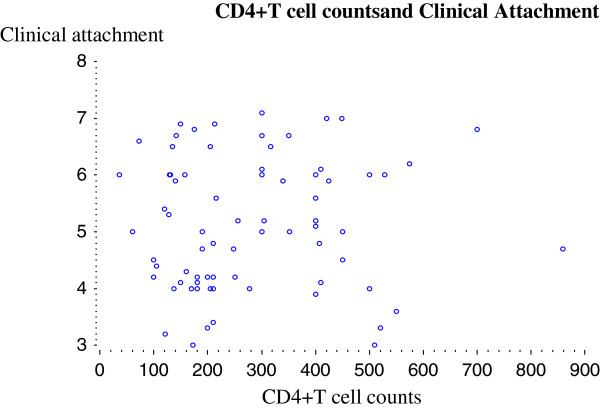
**Correlation of CD4 + T cell counts with clinical attachment level.**

Although, plaque and gingival indices showed positive (0.01514) and negative (−0.01133) correlations with CD4 + T cell counts, no significant associations were reflected in the p values, (Table 2). However, significant correlations were indicated between clinical indices (PD, CAL) and the total CD4+ count (A + B + C). Figure 2 demonstrates the positive Spearman’s correlation between clinical attachment level and CD4 + T cell counts. The lack of a correlation between individual groups A, B and C, with any of the clinical indices (Table 2), shows that the prevalence of periodontal disease in HIV-positive patients may not directly be related to the different stages of immunosuppression even though total CD4+ counts could be associated with changes observed in the periodontal measurements.

**Table 2:** **Association of clinical indices with CD4** + **T cell counts**

**Index**	**Grouped CD4+ T cells/mm**^**3**^			**Levels of association**	
	**A (<200) n = 36**	**B (200-500) n = 71**	**C (>500) n = 13**	**CD4 + T cell Groups (A, B, C)**	**Total CD4 + T cells (A + B** + **C) n = 120**
PI*					
Mean (SD)	2.59 (0.49)	2.52 (0.55)	2.59	rho = −0.02748	rho = 0.01514
Median	2.9	2.8	(0.56)	p = 0.7657	p = 0.8696
Min-Max	1.3-3.0	0.8-3.0	2.8		
			1.2-3.0		
GI					
Mean (SD)	2.8 (0.36)	2.7 (0.48)	2.7 (0.52)	rho = −0.03339	rho = −0.01133
Median	3.0	3.0	3.0	p = 0.7173	p = 0.9023
Min-Max	1.9-3.0	0.5-3.0	1.4-3.0		
PD					
Mean (SD)	4.6 (0.95)	4.83 (1.07)	4.86 (1.17)	rho = 0.09761	rho = 0.18472
Median	4.16	4.9	5.0	p = 0.2889	p = 0.0434
Min-Max	2.9-6.4	3.0-6.8	2.9-6.5		
CAL					
Mean (SD)	5.09 (1.06)	5.37 (1.07)	5.44 (1.36)	rho = 0.12376	rho = 0.20212
Median	5.0	5.5	5.9	p = 0.1781	p = 0.0268
Min-Max	3.0-6.9	3.3-7.3	3.0-7.0		

*PI = Plaque index, *GI* = Gingival Index, *PD* = Probing depth, *CAL* = Clinical loss of attachment.

### Influence of age on clinical indices

With an age range of 20–55 years (Table 1), we used Spearman rank correlation to examine whether age influenced the measurement of periodontal indices. Age was related to the clinical indices regardless of the patients’ CD4 + T cell counts or their stages of immunosuppression. A highly significant positive correlation was found between age and plaque index (p = 0.0018) with a significant correlation with gingival index and probing depth (Table 3). However the level of clinical attachment showed no significant correlation with age.

**Table 3:** **Correlation of age and CD4**+ **T cell counts with clinical indices**

	**Correlation**	**Plaque index**	**Gingival index**	**Probing depth**	**Clinical attachment loss**
Age	rho	0.28149	0.18813	0.18842	0.15461
	p value	0.0018	0.0396	0.0393	0.0918
HIV stage (Groups A, B, C)	rho	−0.02748	−0.03339	0.09761	0.12376
	p value	0.7657	0.7173	0.2889	0.1781
Total CD4 + T cell counts (A + B + C)	rho	0.01514	−0.01133	0.18472	0.20212
	p value	0.8696	0.9023	0.0434	0.0268

p = <0.05.

### Influence of HIV stage on clinical indices

Significant associations were observed between total CD4 + T cell counts (A + B + C) and probing depth (p = 0.0434) and between total CD4+ T cell counts and clinical attachment loss (p = 0.0268), but when the counts were grouped according to HIV stage (A, B and C), these associations were no longer evident (Table 3).

### Influence of smoking on clinical indices

A noticeable but not significant difference was observed between smokers and non-smokers for total CD4+ counts (Table 4) but not for HIV stage (data not shown), although significant differences were observed between smokers and non-smokers for all of the clinical indices, with the exception of the gingival index (Table 4).

**Table 4:** **Influence of smoking on CD4**+ **counts and clinical indices**

**Smoking**							
	**Yes (n = 60)**			**No (n = 60)**			
**Variables**	**Mean (SD)**	**Median**	**Minimum-Maximum**	**Mean (SD)**	**Median**	**Minimum-Maximum**	**p value**
CD4 + T cell counts	322.45 (164.46)	312	61-859	264.4 (131.4)	214	36-562	0.0540
Plaque index	2.66 (0.49)	2.9	0.8-3	2.44 (0.56)	2.55	1.2-3	0.0180
Gingival index	2.8 (0.45)	3	0.5-3	2.70 (0.45)	3	1.4-3	0.1500
Probing depth	4.99 (0.9)	5	3-6.8	4.54 (1.04)	4.15	2.9-6.4	0.0191
Clinical attachment level	5.55 (1)	5.75	3.4-7.3	5.03 (1.13)	5	3-7.1	0.0109

(p < 0.05).

### Association between HIV stage and anti-retroviral therapy (ART)

A greater percentage of HIV patients on ART belonged to Group B, while many of the Group A patients were not on ART (Table 5). Pearson’s chi-square test indicated a highly significant difference between HIV stage and ART (Table 5).

**Table 5:** HIV stage relative to ART

**ART**	**Grouped CD4 + T cell counts (A,B,C) (p = <0.0001)**			**Total**
	A (<200)	B (200–500)	C (>500)	
Negative	32 (68.1%)	14 (29.8%)	1 (2.2%)	47 (39.2%)
Positive	4 (5.5%)	57 (78.1%)	12 (16.5%)	73 (60.9%)

### Influence of ART on clinical indices

The median for CD4+ T cell counts of patients on ART was higher than for those not on ART, with a highly significant positive relationship found between total CD4 + T cell counts and ART (Table 6). ART significantly influenced probing depth (p = 0.0065) and clinical attachment level (p = 0.0029), while no significant relationships were found between ART and plaque index, nor between ART and gingival index.

**Table 6:** **CD4**+ **T cell counts and clinical indices relative to ART**

**ART**							
	**Yes (N = 73)**			**No (N = 47)**			
**Variables**	**Mean (SD)**	**Median**	**Min-Max**	**Mean (SD)**	**Median**	**Min-Max**	**Spearmans correlation (p value)**
CD4 + T cell counts	377.74 (126.97)	352	140-859	162.47 (70.67)	158	36-520	0.77004 (<.001)
Plaque index	2.58 (0.53)	2.8	0.8-3	2.51 (0.55)	2.8	1.3-3	0.03499 (0.7044)
Gingival index	2.75 (0.48)	3	0.5-3	2.76 (0.4)	3	1.8-3	−0.01551 (0.8665)
Probing depth	4.97 (1)	5	2.9-6.8	4.45 (1)	4	2.9-6.4	0.24716 (0.0065)
Clinical attachment	5.53 (1.03)	5.6	3-7.3	4.92 (1)	4.6	3-6.9	0.26956 (0.0029)

### Influence of oral health care on clinical indices

When questioned about frequency of visits to the dentist, 19% of the cohort reported never seeing the dentist, 52% said they saw a dentist once in 5 years and the remainder (29%) claimed they had visited the dentist 2 or more times in the past 5 years.

The majority (70%) reported brushing once a day, while only 30% brushed twice a day. Only 26% reported using interdental aids. A significant difference (p = 0.0352) was observed for plaque index scores of patients who brushed twice a day compared with those who brushed once a day. None of the other clinical indices showed any correlation with frequency of brushing (Table 7). With the exception of probing depth, all the clinical indices were significantly associated with the use of interdental aids (Table 7). The association of clinical indices with CD4+ counts was also significantly improved with adequate oral hygiene practices (Table 7).

**Table 7:** **Clinical indices relative to oral health care and CD4 counts** (**Wilcoxon test**)

**Oral care**		**Plaque index**	**Gingival index**	**Probing depth**	**Clinical attachment loss**	**CD4 + T counts**
**Brushing frequency**						
once a day	Mean (SD)	2.63 (0.4)	2.79 (0.4)	4.86 (1.0)	5.39 (1.1)	313.14 (152.63)
n = 84	Median	2.9	3.0	5.0	5.6	312
	Minimum-maximum	1.2-3.0	1.4-3.0	2.9-6.8	3.0-7.3	61-859
twice a day	Mean (SD)	2.38 (0.58)	2.67 (0.53)	4.55 (1.0)	5.06 (1.0)	247.45 (138.7)
n = 36	Median	2.4	2.9	4.15	5.0	213
	Minimum-maximum	0.8-3.0	0.5-3.0	3.0-6.8	3.3-7.0	36-700
Significance	p =	0.0352	0.0911	0.1376	0.1101	0.0190
**Use of interdental aids**						
	Mean (SD)	2.279 (0.6)	2.57 (0.59)	4.45 (1.17)	4.89 (1.12)	231.35 (142.28)
Yes	Median	2.2	2.85	4.0	4.6	211.5
n = 26	Minimum-maximum	0.8-3.0	0.5-3.0	3.0-6.5	3.3-6.9	36-700
No	Mean (SD)	2.63 (0.49)	2.8 (0.39)	4.86 (0.99)	5.3 (1.07)	310.6 (149.6)
n = 94	Median	2.9	3.0	5.0	5.6	308
	Minimum-maximum	1.2-3.0	1.4-3.0	2.9-6.8	3.0-7.3	90-859
Significance	p =	0.0110	0.0102	0.0762	0.0336	0.0170

p = <0.05.

## Discussion

HIV-AIDS is characterised by a profound immunodeficiency resulting from the depletion of CD4+ T helper lymphocytes. Thus CD4+ cell counts are used to stage HIV-AIDS and to initiate antiretroviral therapy [17]. Earlier studies report that 90% of HIV-positive individuals present with oral manifestations of disease [15] including necrotizing ulcerative gingivitis and periodontitis [21]. There is however, no consensus on the association of HIV with periodontal status [22-24]. The issue appears to be clouded by confounding factors such as the level of immunosuppression (HIV stage) and other risk factors for HIV-associated periodontitis [25]. Community periodontal index of treatment needs (CPITN) scores were not used in this study, since previous studies considered it to overestimate the prevalence and severity of periodontal attachment loss among younger individuals, while underestimating them in the elderly [26,27]. In CPITN or CPI (Community Periodontal Index), the mouth is divided into sextants and index or all teeth are examined for the presence or absence of periodontal pockets, calculus and gingival bleeding and the highest score for each sextant noted. Although easy to use and therefore frequently used in epidemiological studies [28], limitations include the lack of measurement of tooth mobility and attachment loss [29,30] , which, along with probing pocket depth, are considered by most epidemiologists as being good indicators of periodontal disease. In addition, CPITN also assumes that a correlation exists between the presence of calculus and periodontal inflammation; an assumption which has been questioned [26,27]. The epidemiological validity of the Ramfjord teeth in representing the periodontal status of the whole mouth has previously been established [23-28]. Although we are mindful of the limitations of partial mouth measurements such as the underestimation of both the extent and prevalence of periodontal disease reported in some studies [27,31] and the bias reported in others [29,30,32], with measurement of sites on the buccal side of the tooth reported to show better reliability than measurement on the lingual side because of better visibility to the examiner, assessment of the Ramfjord teeth was found to reduce time, cost, patient, and examiner fatigue, while also providing a practical alternative to the 168 measurements for each clinical parameter required to characterise the prevalence and severity of periodontal disease in a single whole mouth using full mouth assessment [27].

This is one of very few studies which have attempted to associate HIV stage with periodontal indices, particularly in developing countries. A study by Vastardis et al. [25] examined for an association of periodontal indices with stages of HIV infection. They determined that for individuals with moderate or severe immunosuppression (CD4 + T cell count <500 cells/mm^3^), a significant positive correlation existed with modified gingival index and bleeding index, with no significant correlation with clinical attachment level (p = 0.0560). Unlike the present study, they, along with other researchers [24] could not find any significant association between periodontal indices and CD4 + T cell counts for all the individuals examined. Moreover only 39 patients were used in their study compared to the present study with a sample size of 120. Robinson et al. [32] also reported an association of clinical attachment destruction with progressive HIV infection but not with probing depth. It is generally expected that the lower the immunosuppression of an individual, the higher the severity of periodontal disease detected [21]. In the present investigation, patients were divided into three groups on the basis of their HIV stage as depicted by their CD4+ counts, namely, A (<200 cells/ mm^3^), B (200-500 cells/mm^3^) and C (>500 cells/mm^3^). Although moderate to severe forms of periodontal disease were observed with the majority of individuals presenting with probing depths and clinical attachment levels > 5 mm, no significant associations were found between any of the periodontal indices and HIV stage. However, when examining the entire HIV + cohort, significant associations were observed between CD4+ counts and probing depth (p = 0.0434) and CD4+ counts and clinical attachment level (p = 0.0268). A relationship existed between the immunosuppression of the study group and their periodontal status, but the level of immunosuppression did not appear to favour the severity of periodontal disease. These findings are similar to those of other researchers who found periodontal disease to be less prevalent in subjects with CD4+ counts < 200 cells/mm^3^ than in subjects with CD4+ counts > 500 cells/mm^3^[26,32-35]. They observed linear gingival erythema (LGE) and necrotizing ulcerative gingivitis (NUG) in patients comparable to our group B only but not the A nor C groups. They also reported that necrotizing ulcerative periodontitis (NUP) occurred with similar prevalence in groups A and B, but not in C. These studies support the suggestion that the use of antiretroviral therapy (ART) has modified the prevalence and course of periodontal disease in HIV-positive patients [12,13,35,36] with reduced incidence of periodontal damage [37-39].

Other confounding factors such as age, smoking and oral hygiene practices were also investigated in the present study. There were significant positive relationships found between gingival index (p = 0.0396) and probing depth (p = 0.0393) with the age of the study population. Plaque index also showed a highly significant (p = 0.0018) positive relationship with age. The age range of the study population (20–55 years) may support the fact that as the individual ages, the chances of developing periodontal disease are increased [16]. A study by Yalcin et al. [40] reported no association between clinical parameters and age.

Smoking showed a marked although not significant association with CD4+ cell counts (p = 0.0540), while being significantly related to plaque index, probing depth and clinical attachment loss. Hence, the present study confirmed smoking as a major risk factor for periodontal disease as found in previous studies [41-43] and highlighted the advantage of smoking cessation in improving the oral health and quality of life of HIV-positive patients [44-46].

It is generally accepted that good oral hygiene is essential in maintaining a disease-free mouth. The present study included patients who had not received dental treatment 3 months prior to the study, thus increasing their chances of developing periodontal disease. When the periodontal clinical indices were related to the oral hygiene practices, the frequency of brushing was found to be significantly associated with plaque index (p = 0.0352) but not with the other periodontal indices. However, the use of interdental aids showed significant associations with all of the periodontal indices except the probing depth. These results clearly suggest that although increased frequency of brushing may have reduced the initial plaque accumulation, the additional use of interdental aids provided better plaque control and improved gingival health. Moreover, only 10.83% of the individuals managed regular dental visits. This may be due to a host of factors including a general lack of interest for maintaining better oral hygiene, lack of access to medical and dental care or other factors beyond their control.

A significant association was established between HIV stage and brushing frequency (p = 0.0190) as well as the use of interdental aids (p = 0.0170). These results reveal that the clinical signs and symptoms of gingival and periodontal disease with reduced CD4 + T cell counts remain a significant complication of HIV infection.

In selecting the study group, no consideration was given to whether patients were on antiretroviral therapy or not, since no consistency of the effect of ART on periodontal disease progression has ever been demonstrated [47]. Because different stages of HIV were being compared, it was considered outside of the scope of this study to include a healthy HIV-negative control group. A direct comparison of this to other studies was complicated by the lack of data reported, including the use and duration of antiretroviral therapy and adjunctive antimicrobials, the broad reference in the literature to HIV- positive subjects without referring to their HIV stage, and the lack of mention of confounding factors such as age, smoking, oral hygiene and other risk factors for periodontal diseases.

## Conclusion

This study established an association between CD4 + T cell counts and chronic inflammatory periodontal disease in HIV-positive patients regardless of their HIV stage. The high prevalence of periodontal manifestations underlines the need for proper home care, appropriate periodontal treatment and maintenance to provide reasonably good periodontal health among HIV-positive individuals. Although ART significantly influenced PD and attachment levels in these HIV-positive patients, the findings of this study conclude that their susceptibility to disease is largely determined by their oral hygiene and smoking habits rather than age, ART or HIV stage.

## Abbreviations

AIDS: Acquired immune deficiency syndrome; ART: Anti-retroviral therapy; CD4+ T cells: Cluster of Differentiation 4 T Helper Lymphocytes; CIPD: Chronic inflammatory periodontal disease; CPITN: Community periodontal index of treatment needs; HIV: Human immunodeficiency virus; LGE: Linear gingival erythema; NUG: Necrotising ulcerative gingivitis; NUP: Necrotising ulcerative periodontitis.

## Competing interests

The authors declare that they have no competing interests.

## Authors’ contributions

CNJ carried out the clinical study, collected the data for analysis and drafted the manuscript. LXGS participated in the design of the study and supervised the clinical work. CWJA contributed to the conceptual design, interpretation of data, funding of the project, writing of the manuscript. All authors read and approved the final manuscript.

## Authors’ information

CNJ: Recently completed the degree MSc Oral Medicine and Periodontology at the University of the Western Cape and accepted a position at a University in Oman. LXGS (PhD, BChD) is Professor and Head of the Department of Oral Medicine and Periodontology in the Faculty of Dentistry at the University of the Western Cape. CWJA (PhD Med., MSc Dent) is Professor and Head of the Medical Microbiology Cluster in the Department of Medical Biosciences, University of the Western Cape.

## Pre-publication history

The pre-publication history for this paper can be accessed here:

http://www.biomedcentral.com/1472-6831/13/69/prepub
